# Hyperestrogenism Affects Adult Height Outcome in Growth Hormone Treated Boys With Silver-Russell Syndrome

**DOI:** 10.3389/fendo.2018.00780

**Published:** 2018-12-21

**Authors:** Kjersti Kvernebo-Sunnergren, Carina Ankarberg-Lindgren, Karin Åkesson, Mats X. Andersson, Lena Samuelsson, Lovisa Lovmar, Jovanna Dahlgren

**Affiliations:** ^1^Department of Pediatrics, Ryhov County Hospital, Jönköping, Sweden; ^2^Department of Pediatrics, Göteborg Pediatric Growth Research Center, Institute of Clinical Sciences, The Sahlgrenska Academy, University of Gothenburg, Gothenburg, Sweden; ^3^Division of Pediatrics, Department of Clinical and Experimental Medicine, Linköping University Hospital, Linköping, Sweden; ^4^Department of Biological and Environmental Sciences, University of Gothenburg, Gothenburg, Sweden; ^5^Department of Clinical Pathology and Genetics, Sahlgrenska University Hospital, Gothenburg, Sweden

**Keywords:** small for gestational age, estrogens, puberty, mass spectrometry, hypogonadism, gonadotropins, Silver-Russell syndrome, adult height

## Abstract

**Background:** Intrauterine growth retardation and short stature are common features in Silver-Russell syndrome (SRS). Despite recombinant growth hormone (rGH) treatment, poor pubertal height gain, affecting adult height (AH), is common. This study investigated whether growth patterns and estrogen concentrations are associated with AH outcome in rGH treated SRS males.

**Methods:** In this retrospective longitudinal single-center study, 11 males with SRS were classified as non-responders (*NR* = 6) or responders (*R* = 5), depending on AH adjusted for midparental height. Epigenetic analysis and longitudinal growth measures, including bone age, rGH related parameters, pubertal development, gonadotropins and estrogen concentrations, were analyzed until AH.

**Results:** Pubarche before 9 years was only observed in one NR. At 10 years of age, there was no difference in gonadotropins between NR and R. However, estradiol (E2) concentrations at 10 years of age showed a strong association to AH adjusted for MPH (*r* = −0.78, *p* < 0.001). Serum E2 (pmol/L) was significantly higher in NR at ages 10 years [median (range) 2 (<2–5) vs. <2 (<2)], 12 years [23 (10–57) vs. 2 (<2–2)] and 14 years [77 (54–87) vs. 24 (<2–38)] but not at 16 years. Birth weight standard deviation score (SDS) was lower in NR [−4.1 (−4.7 to −2.1) vs. −2.7 (−3.3 to −1.7)]. Weight gain (SDS) until pubertal onset was greater in NR [2.4 (1.4–3.5) vs. 0.8 (−0.4 to 1.7)] and pubertal height gain (SDS) was lower in NR [−1.0 (−2.7–0.4) vs. 0.1 (−0.1 to 1.1)]. At AH, a number of NR and R had high E2 concentrations and small testes.

**Conclusion:** Increased E2 concentrations at age 10, 12, and 14 years were associated to less pubertal height gain, thus affecting AH. Due to the small number of patients, the results need to be confirmed in larger cohorts. The finding of impaired testicular development stresses the need of hormonal evaluation as a complement to clinical and radiological assessment when predicting AH in males with SRS.

## Introduction

Silver-Russell syndrome (SRS) is a rare syndromic growth disorder characterized by intrauterine growth restriction, relative macrocephaly, prominent forehead, hemihypotrophy, and a variety of minor malformations including increased risk of hypospadias in boys (1–4). A diagnosis of SRS is based on clinical observations, and a consensus statement offering recommendations on diagnostic approach and management was published in 2017 (2). Epigenetic changes such as 11p15 and maternal uniparental disomy of chromosome 7 are found in some but not all individuals (5). Untreated, adult height (AH) in SRS males is reported to be −3.7 standard deviation score (SDS) (3). Most children with SRS do not suffer from growth hormone deficiency (GHD) but nevertheless benefit from recombinant GH (rGH) treatment, gaining 1.2–1.4 SDS (6–8). Although SRS is a rare disorder, children with SRS in several aspects are a subgroup of children born small for gestational age (SGA) and may serve as a model for how intrauterine and post-natal growth is associated to height gain and hormonal changes later in life.

Early or premature adrenarche has been reported to be more frequent in the SRS population than in the general population (9). Although age at pubertal onset occurs within the normal range, children with SRS seem to enter puberty at the younger end of the spectrum (2). Furthermore, clinical observations claim that SRS children progress through puberty faster than normally expected (2). Regardless of rGH treatment, there seems to be a steeper decline in height SDS from pubertal onset to AH in SRS patients than that in non-SRS patients born SGA with a trend toward a greater height gain in patients with materna uniparental disomy of chromosome 7 and clinical SRS than in 11p15 (6). One explanation for this may be the effects of sex steroids and in particular estrogens on skeletal maturation, leading to pubertal growth spurt at low concentrations in early puberty and growth plate closure at higher estradiol (E2) concentrations in late puberty (10–13).

The hypothesis of this study was that impaired pubertal height gain affecting AH is associated to increased estrogen concentrations before pubertal onset. We evaluated the associations between growth-patterns from birth, GH-related parameters, pubertal development, prepubertal and pubertal gonadotropin, and estrogen concentrations as well as AH outcome in rGH treated boys with SRS.

## Materials and Methods

### Study Population

Nineteen consecutively referred males born 1988 to 2004 diagnosed with SRS were identified at the national Swedish Centre of reference for SRS at Queen Silvia Children's Hospital in Gothenburg. Eleven of those were longitudinally followed and had reached AH, representing the study subjects. All patients were Caucasian, which was likely due to the demographic of the Swedish population at the time. GH status had been evaluated by both arginine-insulin-tolerance test (AITT) and a 12- to 24-h spontaneous GH secretion test and GHD was defined with a level below a cut-off value corresponding to <10 μg/L, using monoclonal 22 kDa GH-antibodies (14). In one patient referred to the NR group, only a GH spontaneous secretion profile was performed, and in one patient referred to the R group, only an AITT was performed; in both cases with normal results. All subjects had been treated with rGH from an early age (2–6 years) until AH due to SGA and/or GHD indication. When re-evaluating the SRS diagnosis according to current diagnostic standards (2), all patients had ≥4 Netchine-Harbison scores; Tables 1, 2.

**Table 1:** Patient characteristics for non-responders (NR).

**Study number**	**3**	**5**	**6**	**8**	**9**	**11**
NR/R	NR	NR	NR	NR	NR	NR
GA (weeks)	41.9	33.0	35.1	40.3	41.0	32.6
NH score	4/6	5/6	5/6	6/6	6/6	5/6
Epigenetic findings	0	11p15 LOM	0	11p15 LOM	11p15 LOM	0
BW SDS	−2.1	−4.1	−4.1	−4.7	−4.7	−3.7
BL SDS	−2.8	−1.8	−4.2	−4.4	−5.3	−5.1
HCSDS	−1.5	−0.7	−2.5	0.6	−1.8	−1.2
Surgery	Yes^a^	No	No	Yes^b^	No	Yes^d^
Other medical conditions	No	No	No	No	No	No
GHD	Yes	Yes	Yes	No	No	No
GnRH analog	No	No	No	No	No	Yes^e^
Pubarche(years)	11.4	12.5	11.2	11.0	9.4	7.7
Pubertal onset(years)	10.9	11.6	11.0	11.0	11.4	9.6
Testicular volume at AH (mL)	20	12	20	8	8^c^	15^f^
Age at AH(years)	15.3	15.7	16.0	16.6	15.4	15.5
Years on rGH treatment	12.1	13.2	13.0	12.7	10.7	11.6
MPH SDS	−0.3	0.6	−0.2	1.1	0.6	−0.8
Diff SDS	−1.4	−1.7	−1.5	−1.1	−3.2	−1.6

AH, adult height; BL, Birth length; BW, Birth weight; GA, Gestational age; GHD, Growth hormone deficiency; GnRH, gonadotropin–releasing hormone; HC, Head circumference; LOM, loss of methylation; MPH, Midparental height; NR, Non-responder; R, Responder; rGH, recombinant growth hormone; SDS, Standard deviation score; NH, Netchine-Harbison.

a Bilateral orchiopexy because of cryptorchidism.

b Hemihypotrophy with epiphysiodesis at 14 years of age and height 170.6 cm. AH 173.6 cm. There was still 4-cm difference between the length of the legs at AH.

c Reduced from 12 mL at maximum testicular volume.

d Surgery, due to penile hypospadias. Bilateral inguinal hernia that did not require surgery.

e Gonadotropin releasing analog treatment from 11.5 to 12.5 years of age.

f *Reduced from 18 mL at maximum testicular volume*.

**Table 2:** Patient characteristics for responders (R).

**Study number**	**1**	**2**	**4**	**7**	**10**
NR/R	R	R	R	R	R
GA (weeks)	35.4	36.6	39.7	37.6	38.0
NH score	5/6	6/6	5/6	4/6	4/6
Epigenetic findings	11p15 LOM	11p15 LOM	11p15 LOM	0	0
BW (SDS)	−2.5	−3.1	−3.3	−1.7	−2.7
BL (SDS)	−2.8	−3.5	−2.2	−0.9	−2.4
HC (SDS)	−0.4	−0.1	−1.5	−0.2	−1.2
Surgery	No	Yes^a^	No	No	Yes^c^
Other medical conditions	No	Yes^b^	No	Yes^b^	Yes^d^
GHD	No	No	No	Yes	No
GnRH analog	No	No	No	No	No
Pubarche(years)	12.5	11.9	13.6	13.2	16.0
Pubertal onset(years)	10.5	12.5	11.5	12.1	15.1
Testicular volume at AH (mL)	22	20	8	17	20
Age at AH(years)	17.5	17.0	14.9	18.0	16.7
Years on rGH treatment	14.3	14.0	13.0	11.8	14.2
MPH (SDS)	0.3	0.1	−0.1	−1.0	−1.7
Diff (SDS)	−0.1	−0.8	−0.2	0.5	0.4

AH, adult height; BL, Birth length; BW, Birth weight; GA, Gestational age; GHD, Growth hormone deficiency; GnRH, gonadotropin-releasing hormone; HC, Head circumference; LOM, loss of methylation; MPH, Midparental height; NR, Non-responder; R, Responder; rGH, recombinant growth hormone; SDS, Standard deviation score; NH, Netchine-Harbison.

a Hemihypotrophy with elongation after AH.

b Maternal preeclampsia.

c Unilateral hernia with incarceration at 6 weeks of age.

d *Treatment for hypothyroidism from 5 years of age*.

**Figure 1:**
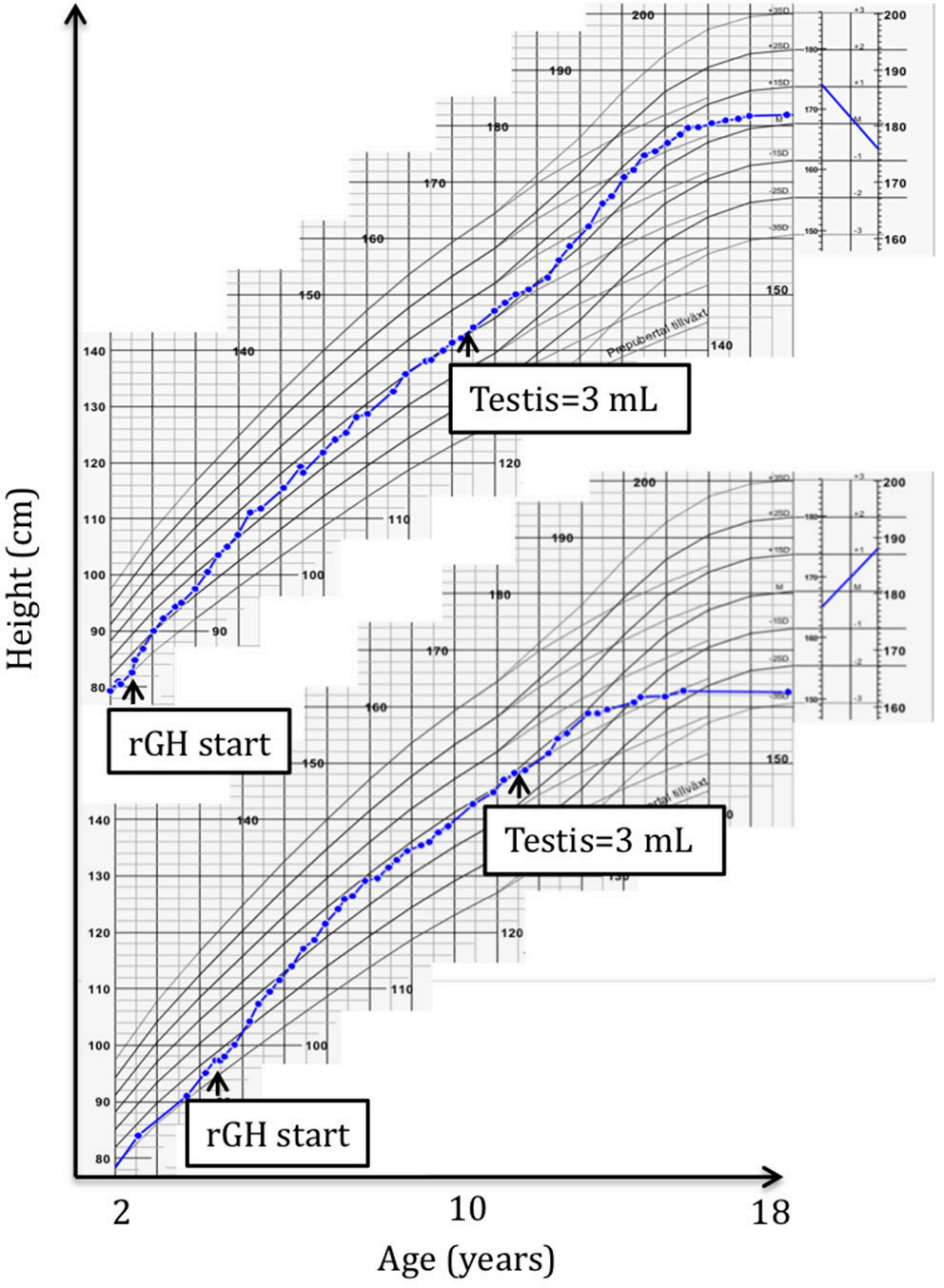
A typical responder (R) height growth chart **(top)** compared to a typical non-responder (NR) height growth chart **(bottom)** (15). Start of recombinant growth hormone (rGH) treatment and pubertal onset are pointed out.

Depending on AH (SDS) adjusted for mid parental height (MPH), participants were retrospectively divided into one of two groups; six subjects with AH (SDS) >1 SDS below MPH were defined as non-responders (NR), and five subjects with AH (SDS) ≤1 SDS below MPH were defined as responders (R). AH (SDS) was calculated as AH in cm converted to SDS at 18 years of age (15) and MPH (SDS) was calculated as the mean of maternal and paternal AH (SDS). According to the European Agency for the Evaluation of Medicinal Products, rGH treatment may be considered in SGA patients without catch-up growth deviating more than −1 SDS from MPH, thus indicating that height within −1 SDS from MPH should be considered normal (16). The definitions of NR and R do not account for the overall Δ height (SDS) gain during rGH treatment *per se*, but the definitions account for whether a height close to MPH was reached by AH. Figure 1 exemplifies growth patterns (15) observed in R and NR. For patient characteristics, see Tables 1, 2.

### Study Protocol

Data on gestational age as well as birth weight (BW), birth length and head circumference were obtained from birth charts and converted to SDS according to Niklasson et al. (17). Blood samples were drawn in the morning (8–11 h) at the start and end of the rGH treatment period and once every year during rGH treatment. After separation, sera were stored at −80°C until analyzed. The stored blood was used to assess changes in epigenetic factors and concentrations of luteinizing hormone (LH), follicle stimulating hormone (FSH), and estrogens at different ages. Other data were collected from patient records. Height, weight and pubertal status were routinely recorded until AH, which was defined as a growth velocity of less than 1 cm/year. Bone age (BA) was assessed from rGH start until 12 years at which age most patients had reached pubertal onset, using standardized routine X-rays that were assessed by a single radiologist using the Tanner-Whitehouse 2 (TW2) method (18). When there were two X-rays equally close to the target age, BA was extrapolated from two sets of samples. Data on the duration of rGH therapy, the individual adjusted rGH dose and the Δ insulin like growth factor-I (IGF-I) (SDS) (19) after 1 year of treatment are shown in Table 3.

**Table 3:** Clinical data [median (range)] for non-responders (NR) and responders (R).

**Parameters**	**NR (*n* = 6)**	**R (*n* = 5)**	*P*-values^*^
**AT BIRTH**			
Gestational age (weeks)	37,7 (32,6−41,9)	37,6 (35,4−39,7)	ns
Birth weight (SDS)	−4.1 (−4.7 to −2.1)	−2.7 (−3.3 to −1.7)	0.045
Birth length (SDS)	−4.3 (−5.3 to −1.8)	−2.4 (−3.5 to −0.9)	ns
Head circumference (SDS)	−1.4 (−2.5 to 0.6)	−0.4 (−1.5 to −0.1)	ns
**AT rGH START**			
Age (years)	3.5 (2.7 to 4.5)	2.9 (2.0 to 6.0)	ns
Weight (SDS)	−4.0 (−5.2 to −2.2)	−4.0 (−5.0 to −3.6)	ns
Height (SDS)	−3.0 (−4.7 to −1.8)	−2.9 (−3.3 to −2.2)	ns
MPH (SDS)	−0.3 (−0.8 to 1.0)	−0.1 (−1.7 to 0.3)	ns
Height (SDS) from MPH	−3.0 (−5.3 to −1.5)	−2.3 (−3.3 to −1.2)	ns
IGF-I (SDS)	−0.2 (−1.6 to 1.0)	−2.8 (−7.0 to 1.4)	ns
**1 YEAR AFTER RGH START**			
rGH dosage (mg/kg/week)	0.28 (0.18 to 0.53)	0.25 (0.20 to 0.45)	ns
Δ IGF-I from rGH start (SDS)	2.4 (1.6 to 3.0)	3.3 (−0.2 to 4.7)	ns
Δ Height from rGH start (SDS)	0.9 (0.4 to 1.3)	0.8 (0.2 to 1.4)	ns
**AT 6 YEARS**			
Weight (SDS)	−2.6 (−4.2 to −0.8)	−2.3 (−4.4 to −1.6)	ns
Height (SDS)	−1.8 (−3.1 to 0.2)	−0.9 (−3.3 to −0.6)	ns
Height (SDS) from MPH	−1.8 (−3.7 to 0.5)	−0.8 (−2.3 to 0.0)	ns
**AT TESTICULAR VOLUME (3 mL)**			
Age (years)	11.0 (9.6 to 12.1)	12.1 (10.5 to 15.1)	ns
Weight (SDS)	−1.0 (−1.8 to −0.6)	−1.3 (−2.7 to −0.7)	0.082
Δ Weight from birth (SDS)	2.4 (1.4 to 3.5)	0.8 (−0.4 to 1.7)	0.013
Δ Height from birth (SDS)	3.0 (1.2 to 4.5)	1.7 (0.3 to 2.9)	ns
Height (SDS)	−0.6 (−1.5 to 0.3)	−0.6 (−2.4 to 0.1)	ns
Height (SDS) from MPH	−0.6 (−1.6 to 0.6)	−0.2 (−0.7 to 0.2)	ns
BMI	15.3 (13.9 to 16.2)	14.7 (14.2 to 17.1)	ns
**At AH**			
Years on rGH treatment	12.5 (11.0 to 14.0)	14.0 (12.0 to 14.0)	0.082
Δ Height during puberty (SDS)	−1.0 (– 2.7 to 0.4)	0.1 (−0.1 to 1.1)	0.045
Height (SDS)	−1.7 (−2.7 to −0.1)	−0.6 (−1.4 to 0.2)	0.068
Height (SDS) from MPH	−1.5 (−3.2 to −1.1)	−0.2 (−0.8 to 0.5)	0.006

* P-values < 0.1 are given in exact measures. Mann-Whitney U test was used for statistical analysis.

*AH, adult height; BMI, body mass index; IGF-I, insulin-like growth factor-I; MPH, Midparental height; NR, Non-responder; R, Responder; rGH, recombinant growth hormone; SDS, Standard deviation score*.

### Pubertal Classification

Signs of early adrenarche defined as pubarche before the age of 9 years were recorded (9). Pubertal stages were classified according to the largest testis using an orchidometer (20), and pubertal onset was defined by a testicular volume of ≥3 mL. This definition was chosen because of substantial evidence indicating that the hypothalamic-pituitary-gonadal (HPG) axis is activated at a testicular volume of 3 mL (21–23).

### Hormone Analysis

Serum LH and FSH concentrations were determined using chemiluminescent microparticle immunoassay (Architect i2000SR, Abbott Scandinavia). Limit of detection (LOD) was 0.1 IU/L for LH and 0.05 IU/L for FSH. Total coefficient of variation (CV) for LH was 7% at 7 IU/L and 50 IU/L and for FSH 6% at 15 and 45 IU/L, respectively. Serum concentrations of estrone (E1) and E2 were simultaneously determined by high-sensitive gas chromatography-tandem mass spectrometry (GC-MS/MS) from Agilent Technologies, Montréal, Canada. LOD was 9 pmol/L for E1 and 2 pmol/L for E2, as previously described (24). Total CV for E1 was 14% for 38 pmol/L and 12% for 100 pmol/L. For E2 the total CV was 19% for 8 pmol/L and 6% for ≥36 pmol/L. Serum IGF-I concentrations were determined by radioimmunoassay (Mediagnost GmbH, Tübingen, Germany). LOD for IGF-I was 0.064 μg/L, and the total CV were 20% and 14% at concentrations of 33 and 179 μg/L, respectively.

### Genetic Analysis

DNA was extracted from peripheral blood lymphocytes, and methylation levels were determined by methylation-sensitive multiplex ligation-dependent probe amplification (MS-MLPA, commercial kit ME30 and ME032 from MRC-Holland; SeqPilot software from JSI Medical Systems GmbH). The ME030 kit targets differently methylated regions on chromosome 11p15, and the ME032 kit targets differently methylated regions on chromosome 6q24, chromosome 7p12.1 and 7q32.2 and chromosome 14q32.2. Methylation levels at each locus were intra-sample normalized and then compared to methylation levels observed in healthy reference samples. A methylation level below 0.5 on chromosome 11p15 was considered indicative of loss of methylation (LOM) (25).

### Statistical Analysis

Data are expressed as median (range), if not stated otherwise. Hormone concentrations below the LOD were set to LOD/2. Statistical comparisons were conducted between groups at ages closest to 6, 8, 10, 12, 14, and 16 years using the Mann-Whitney *U* test. IBM SPSS Software Corp. (USA) (version 24) was used for statistical analysis. Associations between E2 at 10 years of age and AH adjusted for MPH was analyzed by simple linear regression analysis using Pearson's correlation coefficient with 95% confidence intervals. Figures were drawn using Origin 9.0 (OriginLab Corp., Northampton, MA, USA). A *p*-value below 0.05 was considered significant.

### Ethics

This study was carried out in accordance with recommendations of the Regional ethical review board in Gothenburg (449-16). The Regional ethical review board of Gothenburg approved the protocol. Written and informed consent was obtained from the parents and retrospectively from the patients at adult age in accordance with the Declaration of Helsinki.

## Results

Clinical data including genetic findings for the study group are presented as individual data in Tables 1, 2 and grouped data in Table 3.

### Growth Data

BW (SDS) was significantly lower in NR compared to R. Although there was no significant difference in weight or body mass index (BMI) at pubertal onset between the groups, weight gain from birth to pubertal onset was greater in the NR group; Table 3. At the beginning of rGH treatment no significant difference in height (SDS) was observed. Furthermore, height gain during puberty was lower in NR, resulting in impaired AH in NR; Table 3.

### GH and IGF-I Status

GHD was confirmed in one R and three NR; Tables 1, 2. At the beginning of rGH treatment, there was no significant difference in IGF-I (SDS) concentrations between the two groups. Changes in IGF-I (SDS), height (SDS) and weekly rGH dose (mg/kg) at start and after 1 year of treatment, as well as the number of years with treatment, were similar in both groups; Table 3.

### Estrogen Levels Relative to Age and Puberty

**Figure 2:**
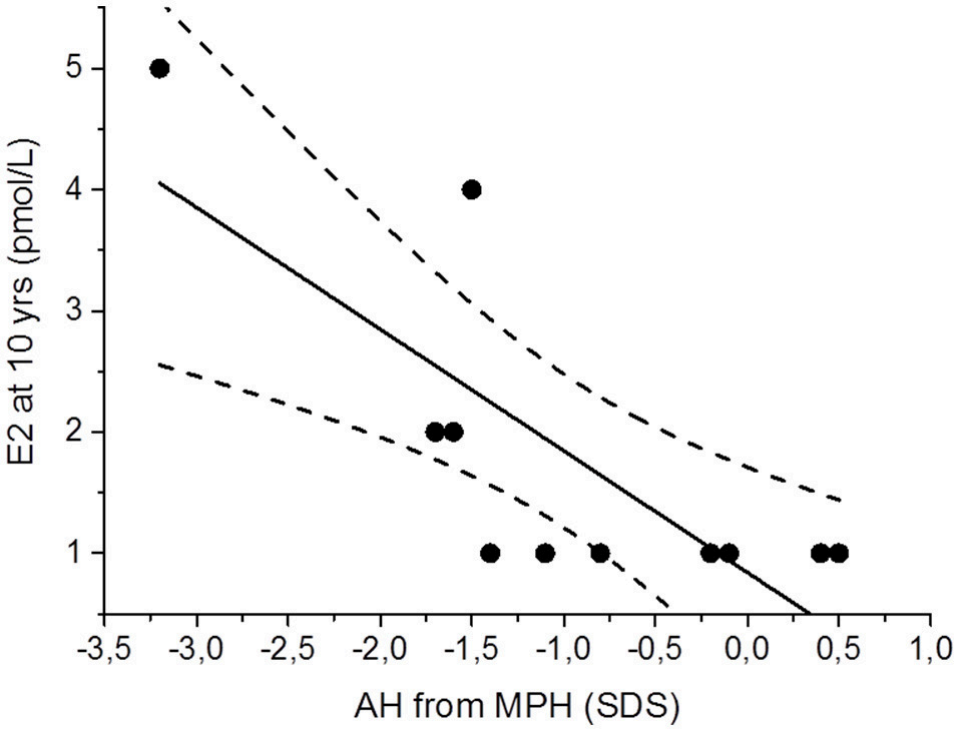
Scatter plot presenting the association between serum estradiol (E2) concentrations at 10 years (yrs) and adult height (AH) adjusted for mid parental height (MPH) in 11 males with Silver-Russell Syndrome. Linear regression analysis (95% confidence bands as dashed lines) yielded a strong negatively correlation: *r* = −0.78, *p* < 0.001.

E2 concentrations at 10 years of age showed a strong correlation to AH adjusted for MPH (*r* = −0.78, *p* < 0.001); Figure 2.

**Figure 3:**
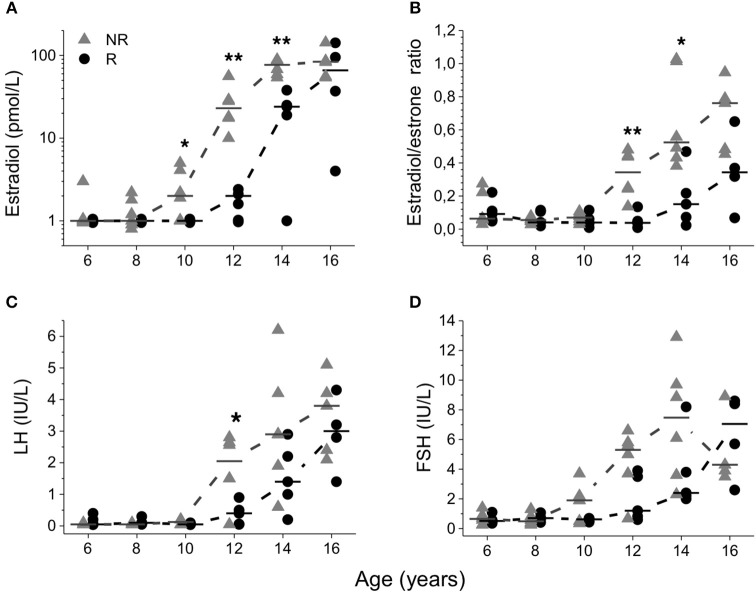
Serum estradiol concentrations **(A)**, estradiol/estrone ratio **(B)**, luteinizing hormone (LH) **(C)**, and follicle stimulating hormone (FSH) **(D)** plotted relative to age. Median values were calculated and denoted as a line for each group and age. Triangles represent non-responders (NR) and circles represent responders (R). Differences between groups are shown as ^*^p < 0.05 and ^**^p < 0.01.

Before 10 years of age, both groups had similar E2 concentrations and E2/E1 ratios; Figures 3A,B. Only one subject in the NR group had a testicular volume of 3 mL at age 10. Nevertheless, the median E2 concentration was higher in the NR group than in the R group [2 (<2–5) vs. <2 (<2) pmol/L; *P* = 0.034] at this age; Figure 3A. This discrepancy between the two groups was even more evident at the ages of 12 years [23 (10–57) vs. 2 (<2–2) pmol/L; *P* = 0.006] and 14 years [77 (54–87) vs. 24 (<2–38) pmol/L; *P* = 0.006]. Moreover, at 12 and 14 years of age, the median E2/E1 ratio was higher in the NR group [0.34 (0.14–0.48) vs. 0.04 (0.01–0.14); *P* = 0.006 and 0.52 (0.38–1.03) vs. 0.15 (0.02–0.47); *P* = 0.018]; Figure 3B. At 16 years of age, samples from 9 patients were available (with one patient missing from each group). At this age, there was a borderline significant difference in the E2/E1 ratio with a higher ratio in the NR group [0.76 (0.45–0.95) vs. 0.34 (0.07–0.65); *P* = 0.050]; however, there were no differences in the E2 concentrations between the two groups; Figures 3A,B.

**Figure 4:**
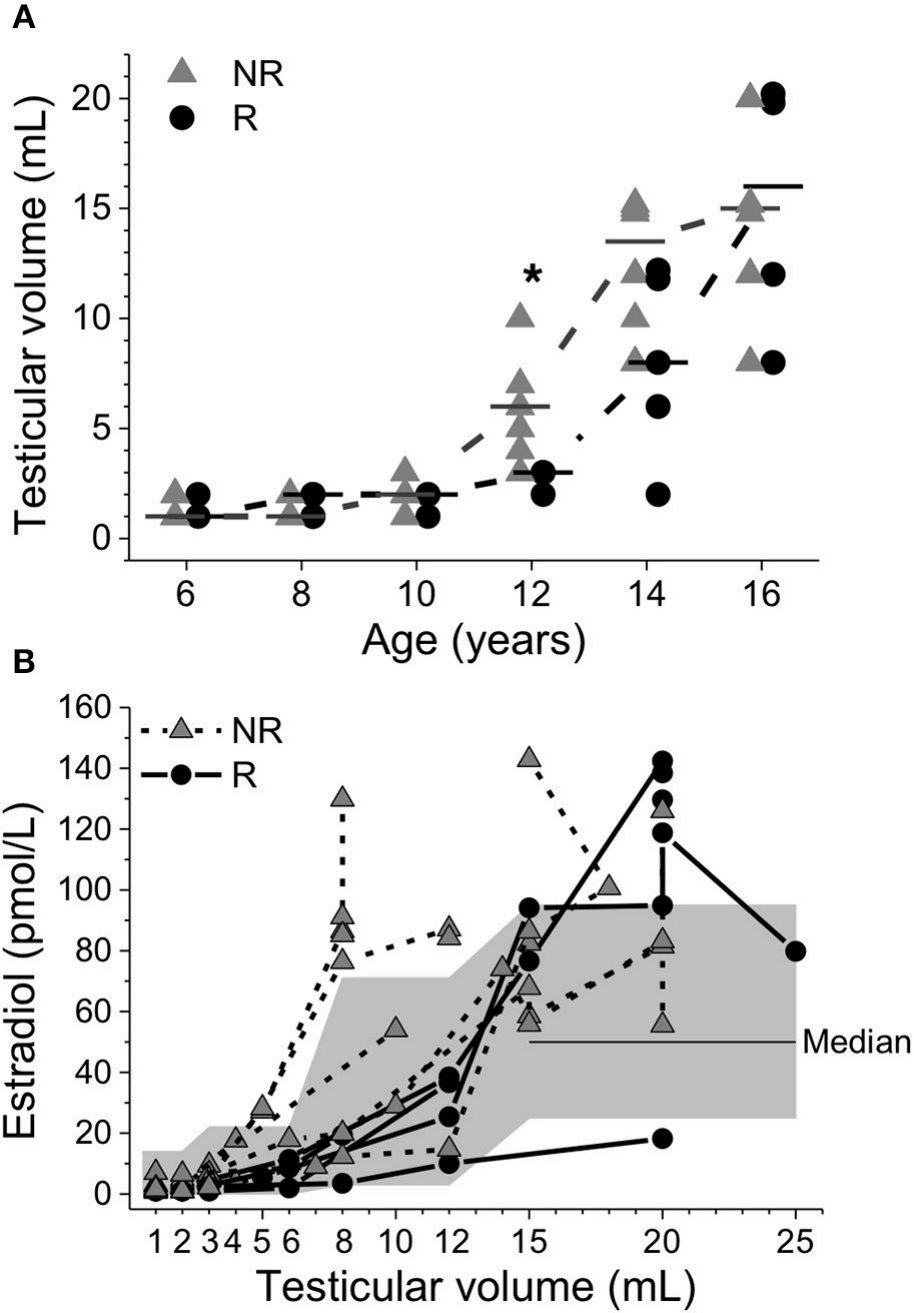
Testicular volume plotted relative to age **(A)**. Median values were calculated and denoted as a line for each group and age. Triangles represent non-responders (NR) and circles represent responders (R). Differences between groups are shown as ^*^p < 0.05. Serum estradiol concentrations plotted relative to testicular volume **(B)**. The gray area depicts reference intervals for healthy boys during pubertal development (24).

Although NR had earlier age at pubarche [11.1 (7.7–12.5) vs. 13.2 (11.9–16.0); *P* = 0.011], pubarche before the age of 9 years was uncommon and only observed in one NR. In two NR and one R, pubarche was observed before onset of puberty. Moreover, although no age difference was seen at pubertal onset between the groups, NR had a significantly larger testicular volume at 12 years of age [6 (3–10) vs. 3 (3–6) mL; *P* = 0.011]; Figure 4A. Furthermore, at 12 years LH (IU/L) was higher in NR [2.1 (0.1–2.8) vs. 0.4 (0.1–0.9); *P* = 0.032] but no significant difference was seen in LH at 10 years in NR 0.2 (<0.1–0.2) vs. *R* < 0.1 (<0.1); Figure 3C. After 12 years of age no significant differences were seen in LH concentrations and no significant differences were seen in FSH (IU/L) concentrations at any age; Figure 3D. At 16 years of age there was one missing data from each group.

Figure 4B shows that beginning at a testicular volume of 5 mL, some NR had E2 concentrations above reference intervals for boys during pubertal development (24). At the end of puberty, patients from both groups, except for one R, had E2 concentrations above the median reference value for adolescent males. At AH, the testicular volume of 1/5 of R and 3/6 of NR did not reach a normal adult testicular volume (≥15 mL). In 2/6 of NR, the testicular volume decreased after the age of 16 years; see Tables 1, 2 for individual data.

### Bone Age

No significant differences in BA were found between NR and R, at age 6, 8 or 10 years [5.0 (3.4–6.9) vs. 4.9 (3.8–5.7); 8.1 (5.0–10.4) vs. 7.3 (6.5–7.9); and 10.8 (7.5–12.3) vs. 9.3 (9.1–10.5) years, respectively]. There were no differences in BA between groups at pubertal onset [13.0 (9.1–13.9) vs. 12.8 (10–14.3) years] or chronological age of 12 years [12 (11.4–14.1) vs. 11.7 (11.5–13.3) years]. X-rays were missing in one NR and one R at 12 years of age, at which point both patients had a testicular volume of 3 mL.

## Discussion

The main finding of this study was the association between E2 concentrations at 10 years of age and AH outcome adjusted for MPH. NR not reaching MPH despite rGH treatment had lower BW and higher E2 concentrations at ages 10, 12, and 14 years. Increased median E2 concentrations in NR were observed two years earlier compared to R. Moreover, the median loss in height of 1.0 SDS during puberty in NR compared to R, who gained 0.1 SDS, indicated that the difference in AH outcome was caused by incomplete pubertal growth spurt in the NR group. At the end of puberty, several patients from both groups had E2 concentrations above the reference interval for healthy adolescent males. Small adult testicular volumes were observed in a number of patients, and some NR even developed reduced testicular volumes at the end of puberty. Differences in variables related to rGH treatment could not explain the impaired AH outcome in NR. To our knowledge, this is the first publication describing estrogen concentrations during rGH treatment in males with SRS.

The effect of estrogens particularly E2, on bone maturation with subsequent fusion of growth plates is well known (10–13). Although both groups had high E2 concentrations at AH, NR had increasing E2 concentrations 2 years earlier than R. The lack of difference in E2 at 16 years of age reflects that the earlier increase in E2 concentrations rather than high E2 concentrations at AH *per se* affect AH outcome. In children with SRS, early delay of BA followed by rapid acceleration at 8 to 9 years of age has been reported (2). No significant differences were observed in BA between the two groups, which may have been due to the small number of patients or the fact that BA is not a reliable predictor of height potential in children born SGA (26). Declining height SDS during puberty has been observed in both untreated and in rGH-treated non-SRS children who were born SGA (27, 28). However, despite rGH treatment SRS patients seem to have a steeper decline in height SDS during pubertal spurt compared to non-SRS patients born SGA (6). The results of this study show that only the subgroup of patients with significantly higher E2 concentrations at ages 10, 12, and 14 years and increased E2/E1 ratios at 12 and 14 years had impaired pubertal growth spurt and negatively affected AH. Although accelerated BA in NR was not found in this study, early estrogen exposure in NR is a probable explanation of impaired pubertal height gain and shorter AH through premature bone maturation (10–13).

In the present study a significantly lower BW and greater weight gain from birth to the beginning of puberty was seen in NR. Central adiposity, low BW and rapid weight gain in early childhood may lead to, decreased insulin sensitivity, increased IGF-I concentrations and higher concentrations of adrenal androgens (29). Furthermore, central adiposity affects aromatase activity converting androgens to estrogens and 17β-hydroxysteroid dehydrogenase converting E1 to E2 (30). Interestingly, IGF-II, showing downregulated expression in patients with 11p15 LOM (4, 5, 31, 32), appears to be a potent inhibitor of aromatase activity (33). Our results show that NR who had a significantly lower BW and greater weight catch-up from birth to puberty compared to R did not have significantly higher BMI or weight at pubertal onset. Hence, we believe that BW rather than weight or BMI at pubertal onset explains the difference in Δ weight until start of puberty.

Increased steroid synthesis in the adrenal- and/or gonadal-gland might contribute to the higher E2 concentrations seen in NR through conversion of androgens to estrogens (30). It has been suggested that children born SGA, due to events occurring during fetal development, may show long-term alterations in the hypothalamic-pituitary-adrenal axis that lead to alterations in adrenal activity with earlier and more aggressive adrenarche (2, 4, 31, 32, 34, 35). Furthermore, Binder et al. recently published a retrospective study concluding that early adrenarche is more frequent in rGH treated boys with SRS, but no compromising effect was seen on AH (9). In our study, however pubarche did not occur until after pubertal onset in most patients and in only one NR before the age of 9 years. On the other hand, other publications report disturbed regulation of the HPG axis associated to BW and postnatal growth. Moreover, although normal pubertal timing was reported in boys born SGA (36), hypogonadism was found in adult SGA males without postnatal catch-up growth (37), suggesting that BW and postnatal growth are associated with gonadal function. The results of the current study indicate impaired gonadal function in a number of patients, at least at the end of puberty. The low LH concentrations seen in both groups until the age of 10 years do not indicate that early activation of the HPG axis is present, although there seems to be a more rapid progression through puberty with significantly earlier age at pubarche, and at 12 years of age, both higher LH concentrations and larger testicular volumes in NR. The higher E2 concentrations and lack of difference in LH concentrations at 10 years of age may be due to the small number of patients or reflect a gonadal dysfunction independent of activation of the HPG axis.

The high E2 concentrations observed in both groups by the end of puberty at adult testicular volumes are similar to references for adult males rather than adolescent males (38, 39). A substantial proportion of patients unexpectedly did not reach normal adult testicular volume, and some NR even exhibited a reduction of testicular volume. Together with the finding of high E2 concentrations these results indicate an element of disturbed gonadal function. Hypogonadism has been reported to be frequently present in SRS males in adult life (40), and might be present already during fetal life, explaining the increased risk of male congenital defects of external genitalia, which is reported in as many as 40% of male patients with SRS (2). Taking this in to account, only assessing testicular volume might underestimate the pubertal stage and is unreliable for estimating pubertal development in this group of patients, as testicular function and volume probably are affected already at earlier age.

Determination of estrogen concentrations in children is challenging because serum concentrations of E2 are very low during childhood. The strengths of this study were however the use of a highly sensitive GC-MS/MS-based method, as well as the longitudinal design with repeated serum sampling enabling the identification of the timing of the changes in estrogen secretion patterns. Moreover, epigenetic changes in our cohort were similar to findings reported in larger cohorts, implying that our cohort is a representative SRS population. The results of this study are interesting considering that despite the small number of patients, we found significant differences in BW, weight gain from birth until pubertal onset, prepubertal and early pubertal estrogen concentrations and pubertal height gain between the groups, suggesting a relationship to AH outcome.

The weaknesses of this study are the limited number of patients and the retrospective design and subgrouping of the patients in two groups based on outcome. However, the results of this study have important clinical implications. First, measurements of E2 concentrations with MS/MS rather than clinical assessment of testicular volumes are necessary in order to identify the timing and pace of the biological maturation process in SRS males. Secondly, the results of this study show that the timing of increased estrogen concentrations in SRS males seems to affect pubertal growth spurt and AH, but this needs to be confirmed in larger cohorts.

In conclusion, less pubertal height gain and impaired AH in the NR group were found to be associated with early increase in E2 concentrations and higher E2/E1 ratio. The discrepancies between testicular volumes and E2 concentrations in NR and R reinforce the need for hormonal evaluation as a complement to clinical examination and radiological assessment in SRS males during childhood and puberty when predicting AH.

## Author Contributions

JD was responsible for care and treatment of the patients. KK-S, CA-L, and JD contributed to the study design. CA-L and MA were responsible for the mass spectrometry analysis. LS and LL were responsible for the epigenetic analysis. KK-S, CA-L, and JD contributed to the interpretation and analysis of data. KK-S wrote the first draft of the manuscript. CA-L, JD, and KÅ contributed to the final writing and revising of the manuscript and checked for important intellectual content. All authors approved of the final manuscript as submitted.

### Conflict of Interest Statement

JD received unrestricted grants from Pfizer. The remaining authors declare that the research was conducted in the absence of any commercial or financial relationships that could be construed as a potential conflict of interest.
